# Environmentally sustainable person–centred care: Occupational therapy students' attitudes, perceptions and self‐perceived preparedness for practice

**DOI:** 10.1111/1440-1630.12998

**Published:** 2024-10-15

**Authors:** Felicity Murray, Ka Yan Hess, Tanya Rihtman

**Affiliations:** ^1^ Occupational Therapy Programme, Department of Sport, Nutrition and Allied Health Professions Oxford Brookes University Oxford UK

**Keywords:** advocacy, climate justice, education, environmental sustainability, ethics, occupational therapy, person‐centred care

## Abstract

**Introduction:**

Climate change threatens the environments in which person‐centred occupational therapy occurs. Environmental sustainability is directly linked with the health and wellbeing of current and future generations, presenting occupational therapists with a unique advocacy and activist role. As practitioners of the future, there is an urgent need to understand students' attitudes, perceptions of, and self‐perceived preparedness for, dealing with environmental determinants of health and intergenerational occupational and climate justice.

**Methods:**

A cross‐sectional exploratory descriptive survey collected United Kingdom (UK) based allied health professional students' attitudes, perceptions, and self‐perceived preparedness for advocating for environmental sustainability in the context of person‐centred care. The survey was distributed to 48 gatekeepers in UK institutions with approved allied health professional training programmes (44 offered occupational therapy); 62 occupational therapy students responded. Descriptive and non‐parametric inferential statistics were used to analyse quantitative data. Text and short answers were analysed qualitatively via inductive content analysis.

**Consumer and Community Involvement:**

The study was co‐designed and implemented with MSc (pre‐registration) occupational therapy students.

**Results:**

Participants (94%) expressed concerns for climate change, with 84% feeling responsible for addressing environmental sustainability in health care. While 64.5% identified climate justice as a top priority, a perceived challenge emerged between person‐centred care and sustainability, with only 18% of participants feeling prepared for environmental sustainability in occupational therapy practice. Participants requested education on personal and professional sustainability practices, as well as collective action. Sharing personal climate change experiences, advocating with family and friends, and facilitating connections for collective action were highlighted as potentially transformative educational tools in this area.

**Conclusion:**

Occupational therapy curricula should address environmental sustainability through pragmatic, critical, and ethical lenses to enhance students' preparedness for this advocacy and activist role. Reflection and continuous professional development for environmentally sustainable practices is recommended.

**PLAIN LANGUAGE SUMMARY:**

Occupational therapists believe that it is important to support people to participate in occupations that matter to them. However, the activities that some people choose to participate in may have negative effects on the environment and the planet. It is important to ensure that when occupational therapists support people in their choices of activities, this does not lead to unequal access to healthy and meaningful occupations of others, now or in the future. That is because occupational therapists also have a responsibility to prevent occupational injustice. Occupational therapy students are the professionals of the future, so it is important to include them in research about this topic. They need to develop skills that allow them to simultaneously make sure that they are delivering person‐centred care, which is not environmentally detrimental and that does not lead to occupational injustice. Making sure that occupational therapists provide person‐centred care while also managing risks of occupational injustice may be seen as a profession‐specific dilemma. In this study, occupational therapy students in the UK completed a survey about their feelings, views, and readiness for managing this dilemma. Results showed that most respondents are concerned about climate change, but do not feel that their occupational therapy education sufficiently prepared them to practise in an environmentally sustainable way. They were asked to propose ideas for addressing this issue, and the article discusses how occupational therapy curricula might be changed in accordance.

**Key points for occupational therapy**

- Support is required to turn individual‐level environmental concerns into action and advocacy in occupational therapy practice.
- Education is required to develop skills in evaluating effectiveness of pro‐environmental action and critiquing individual‐centric practices.
- Self‐awareness, policy analysis, and communication practice may support environmental advocacy and activism skill development.

## 1 INTRODUCTION

Climate change is widely acknowledged as a major threat to human health (Campbell‐Lendrum et al., 2023). This anthropogenic crisis (i.e. environmental change that has been either directly or indirectly caused or influenced by human activity) is an unintended outcome of unsustainable human occupations and post‐industrialist lifestyles (Ikiugu et al., 2015). The World Health Organisation (WHO) (2021) has warned that the impact of climate change on health determinants is expected to cause deaths and damage to essential health‐determining sectors such as agriculture, water, and sanitation. The likely result can be seen as a climate justice issue with the potential of deepening global and humanitarian inequalities, not least with regard to occupational access and choice; individual and population engagement in unsustainable occupations today are limiting and threatening occupations of current and future generations (Sultana, 2022). In light of the impacts of climate change on human health and occupation — and hence occupational justice — the inclusion of environmental sustainability within occupational therapy practice and education is now deemed an ethical professional responsibility (Royal College of Occupational Therapists (RCOT), 2021).

The ethical nature of the interrelationships between occupation, human health, and climate change are increasingly recognised (Wagman et al., 2020). Climate injustice refers to the disproportionately greater negative impacts of climate change on economically disadvantaged and vulnerable individuals and communities driven by wealthier countries and individuals (Sultana, 2022). Climate change poses disproportionately severe threats to the health of the economically disadvantaged and vulnerable (Wagman et al., 2020), introducing a direct relationship between environmental sustainability and occupational justice. Drolet et al. (2020) have proposed the term ‘intergenerational occupational justice’ to describe the inequitable impacts that climate change will have on the occupational rights of future generations, due to the unsustainable lifestyles of current generations. This connection between climate justice and occupational justice underpins the ethical responsibility for occupational therapists to become environmental sustainability advocates (Garcia Diaz & Richardson, 2021; World Federation of Occupational Therapists, 2018), with the adoption of political and activist characteristics to the professional identity of occupational therapists' professional identity (Pollard & Sakellariou, 2014).

According to the World Federation of Occupational Therapists (WFOT) (2018: p17), sustainable occupational therapy practice necessitates (i) exploring social, economic, environmental and spiritual determinants as part of prevention of ill‐health; (ii) empowering and enabling people to be drivers of their wellbeing and impact on communities; and (iii) eliminating waste and use of low carbon alternatives in service delivery. As environmental sustainability advocates, occupational therapists can use a variety of lobbying actions within health systems to facilitate the sustainable meeting of human occupational needs, including actions relating to exploration, empowerment, and elimination, as described above by the WFOT (Dhillon et al., 2010; WFOT, 2018). In this role, occupational therapists should use their understanding of the political influences on human occupations to take deliberate and observable actions to create and protect equitable opportunities for occupational engagement across context and time (Pollard & Sakellariou, 2014; Hart et al., 2021). However, multiple challenges hinder the adoption of sustainable occupational therapy practices. These include a limited evidence base (Lieb, 2020), under‐recognition of the profession's environmental sustainability efforts (Wagman et al., 2020), and lack of clarity of what is meant by the term ‘sustainability’ in the context of occupational therapy practice (Hocking & Kroksmark, 2013; Jenkin et al., 2016). Indeed, the WFOT (2012: p1), noted that, ‘it is the challenge for occupational therapists to enable human development and individual well‐being whilst promoting environmentally sustainable well‐being’.

Furthermore, occupational therapists are human too; they are also subject to potential incongruence between theoretical belief and practical action. This phenomenon has been explored by Argyris, Putnam, and Smith (1985, p.82), who proposed a difference between ideal and actual actions, namely a misalignment between ‘espouse theory’ (theories individuals claim to follow) and ‘theory in use’ (theories that are inferred by actions taken). This difference might explain why occupational therapists remain unsure how to practise sustainably (Seville et al., 2023), despite the World Federation of Occupational Therapy's publication of a profession specific guide for sustainable occupational therapy practice and education more than half a decade ago (WFOT, 2018). According to WFOT principle 5 (developing professional competence for administering occupation‐based interventions to address sustainability issues), ongoing engagement to optimise one's knowledge, skills, and attitudes is an essential aspect of developing professional competence (2018: p36). As this continuous journey begins in pre‐registration occupational therapy education, students' actual and perceived preparedness for this vital responsibility is the foundation from which the gap between ‘espouse theory’ and ‘theory in use’ (Argyris et al., 1985) can be reduced.

This ethical responsibility introduces a need to reconcile between individualist and collectivist views (Komatsu et al., 2019). Person‐centred care is a long‐standing foundational principle within occupational therapy practice, part of the core ethical values and responsibilities for practitioners (American Occupational Therapy Association, 2020; WFOT, 2018). Yet the notion of person‐centeredness has been questioned due to its Western, individualistic ideological origin (Restall & Egan, 2021), with arguments raised that this concept and its associated occupational therapy frameworks disproportionately prioritise individual occupations and are thus inadequate to address the challenges posed by climate change (Gerlach et al., 2018; Taff et al., 2014). Whalley Hammell (2015), among others, highlighted the importance of applying a critical perspective to widely accepted occupational therapy principles. This view is echoed by Komatsu et al. (2019) who espoused that ‘although the independent self has traditionally been a major cornerstone of Western civilization …, rewriting this culturally‐derived concept of self might now be necessary to move towards greater environmental sustainability’ (p1). Even if a professional focus shifts from person‐centredness to collective client‐centredness was to occur, this human‐centric focus still needs to be balanced with caring for the environment as an environmental determinant of health (Hess & Rihtman, 2023).

Professional governing bodies have shown growing recognition of the inclusion of environmental sustainability within occupational therapy education (e.g. RCOT, 2019). More contextualised education about how to practise environmental sustainability in occupational therapy is needed beyond linking with sustainable development goals (Seville et al., 2023; Wagman et al., 2020). Students are the practitioners and advocates of the profession of the future. It is imperative that occupational therapy students are prepared to meet the growing ethical challenges posed by climate change (Hess & Rihtman, 2023); however, students' perspectives about environmental sustainability in their education are currently unknown. Moreover, student occupational therapists' personal views and experience of the climate emergency affect their development of critical self‐awareness and degree of sustainability inclusion in their practice, and thus warrants interrogation (Taylor, 2020).

This study had three aims:

- To explore occupational therapy students' attitudes, perceptions, and self‐perceived preparedness towards the challenge of delivering environmentally sustainable person‐centred care.
- To investigate occupational therapy students' perceptions of the balance between meeting responsibilities for person‐centred care and environmentally sustainable wellbeing.
- To explore students' self‐perceived preparedness and educational needs to meet the potential challenge of achieving balance between the two so that they can fulfil their professional advocacy obligations.

## 2 METHODS

### 2.1 Participants

The study objectives noted above were achieved as part of a larger research project co‐designed with students exploring allied health professional students' attitudes towards climate change and environmental sustainability. For the larger project, participants were recruited from 48 United Kingdom (UK) institutions with approved allied health professional training programmes (Health and Care Professions Council, 2024). Of these 48 institutions, 44 offer Royal College of Occupational Therapists (RCOT) accredited occupational therapy programmes (RCOT, n.d.). The current paper reports only on data from pre‐registration occupational therapy students (*n* = 62), the sole inclusion criterion being current enrolment in any pre‐registration occupational therapy UK programme (*n* = 44 programmes (RCOT, n.d.)).

### 2.2 Instruments

As an emerging area of research, an exploratory design was deemed most suitable to support achievement of the study aims (Swedberg, 2020). A cross‐sectional descriptive online survey was developed for the purposes of this study. The survey comprised four discrete sections to enable achievement of the aims of a wider allied health professional research project; of these sections, data related to occupational therapy students were derived from the following sources:

#### 2.2.1 Public perceptions of climate change and health (The Health Foundation, 2021)

The Public Perceptions of Climate Change and Health is a non‐standardised survey on public attitudes around climate change, health care provision, and the UK health and social care system. The development of this survey was commissioned by the UK Health Foundation to explore views of the general UK public on climate change, health, and sustainable health care and was used with a large, representative sample of over 1800 people (The Health Foundation, 2021). As this is an exploratory survey (as opposed to a validated questionnaire), the use of this instrument was deemed suitable for the current exploratory study.

Two adaptations were made to increase relevance for the current study — (1) replacing the phrase ‘social care’ to ‘allied health professions/professionals’ and (2) inclusion of two items for additional analysis/interest (‘Healthcare workers should initiate discussions about climate change with their patients, even if patients did not bring it up themselves’ and ‘Considering the environmental impact of interventions when offering them to patients, even if this means they are less person‐centred e.g. encouraging people to adopt a more plant‐based diet even if eating meat is a large component of their cultural background’).

#### 2.2.2 Youth Climate Justice Survey (ECO‐UNESCO, 2020)

The Youth Climate Justice Survey was developed by ECO‐UNESCO, an Irish environmental education and youth organisation, to explore how young people feel about climate change and issues of climate justice. It is a non‐standardised survey comprising a range of multiple choice and open‐ended questions related to personal impacts of climate change, barriers to engagement, knowledge, and desired types of support. Only items relevant to the study objectives were used, namely those related to respondents' views regarding ethical dilemmas posed by climate change to professionals, and the perceived importance of climate justice as an issue relative to others imposed by climate change. This instrument is a survey (as opposed to a validated questionnaire) which has been used with large samples of comparative populations relevant to the focus of the current study (ECO‐UNESCO, 2020). As such, in light of the cross‐sectional exploratory nature of this study, items were identified for use due to their relevance to the study objectives.

#### 2.2.3 Non‐validated items

Descriptive survey items were developed specifically for this study to gather demographic and educational data. Two items were derived from the WFOT's (2012) Challenge Statement, namely: ‘To what extent do you agree or disagree with the statement: “It is challenging for HCPs to enable person‐centred care whilst promoting environmentally sustainability”?’ and ‘To what extent do you feel that your university education has prepared you to meet this challenge?’ Additionally, respondents were asked to address the following statement: ‘The education about sustainability I received at university has prepared me to promote/practise sustainability’ as well as provide open‐ended text responses around the education on sustainability students would want to receive. Furthermore, using a 4‐point categorisation, participants were asked to indicate their support for, and opposition to, a variety of actions being introduced in health‐care services which were presented with potential personal impacts.

### 2.3 Procedure

Ethical approval was obtained from Oxford Brookes University, Faculty of Health and Life Sciences Ethics Committee (#HLS/2021/CG/31). An online survey was developed using Qualtrics, comprising the sections and items described above, and was disseminated via gatekeepers at 48 UK institutions that have approved Health and Care Professions Council allied health professions' training programmes (Health and Care Professions Council, 2024); of these institutions, the Royal College of Occupational Therapists accredit 44 occupational therapy programmes across the UK (England: *n* = 35; Northern Ireland *n* = 1; Scotland *n* = 4; Wales *n* = 4). In addition, the recruitment poster was shared on Twitter (also known as X) to facilitate snowball sampling (Moule et al., 2017). An information sheet and reminder of the voluntary nature of participation were embedded within the survey (including an explicit statement that participants could exit the survey at any point). Explicit consent was sought prior to starting the survey. Participants must read through the study information sheet and informed consent form first, then followed by selecting a tick as an indication of their explicit consent before the actual survey. Data collection was entirely anonymous, and downloaded data were stored securely on an encrypted database. Occupational therapy students' responses were extracted to create a dataset that would enable attainment of the specific objectives reported in this paper.

### 2.4 Data analysis

Data were analysed using the IBM SPSS Statistics software version 28 (IBM Corp, 2021). Descriptive statistics (percentage, median, interquartile range [IQR]) were used to analyse ordinal level data relating to respondent demographics and student attitudes. Greene and D'Oliviera's (2005: p209) statistical test decision chart was used to support the identification of suitable statistical tests with consideration of the nature of the available variables and the test intention. Non‐parametric inferential statistical tests were used to explore relationships between variables of interest. Spearman's rank‐order correlation coefficients were calculated to explore whether associations existed between student responses to different questions of interest. Dependent on data distribution, chi‐square or Mann–Whitney *U* tests were employed for non‐parametric between‐group comparisons (independent groups).

Qualitative data obtained from responses towards an open text question (i.e. ‘What could be included in your course curriculum about sustainability that would be useful?’) about desired content in the curriculum addressing environmental sustainability were analysed using inductive content analysis, at the levels of both units of meaning as well as categories (Elo & Kyngas, 2008). This process was led by the co‐first author KH who has experience with this form of qualitative analysis. Participants’ responses were read, and units of meaning and associated categories were identified for two rounds (Elo & Kyngas, 2008). All authors reviewed the identified units of meaning and associated categories at each round as a foundation for discussion, and confirmation of the findings presented.

## 3 RESULTS

### 3.1 Demographics

The survey was completed in full by 62 students, all of whom were enrolled on an approved UK pre‐registration occupational therapy programme. While details of specific programmes of study were not gathered to ensure anonymity, responses were received from occupational therapy students from all four countries (England: *n* = 45; Northern Ireland: *n* = 8; Scotland: *n* = 4; Wales: *n* = 5). Information provided by the RCOT (personal communication, February, 2024) indicated that there are approximately 7500 occupational therapy students enrolled each year across all pre‐registration programmes of study in the UK such that the sample in this study represents less than 1% of the total UK occupational therapy pre‐registration student population. Demographic information is presented in Table 1.

**TABLE 1:** Participant demographic information.

		Undergraduate	Masters	Postgraduate diploma	Total
Age group	18–24	*n* = 15 (24.2%)	*n* = 4 (6.5%)	*n* = 0 (0%)	*n* = 19 (30.6%)
	25–29	*n* = 6 (9.7%)	*n* = 12 (19.4%)	*n* = 2 (3.2%)	*n* = 20 (32.3%)
	30–34	*n* = 5 (8.1%)	*n* = 3 (4.8%)	*n* = 0 (0%)	*n* = 8 (12.9%)
	35–39	*n* = 2 (3.2%)	*n* = 2 (3.2%)	*n* = 0 (0%)	*n* = 4 (6.5%)
	40+	*n* = 6 (9.7%)	*n* = 5 (8.1%)	*n* = 0 (0%)	*n* = 11 (17.7%)
	Total	*n* = 34 (54.8%)	*n* = 26 (41.9%)	*n* = 2 (3.2%)	*n* = 62 (100%)
Country	England	*n* = 20 (32.3%)	*n* = 25 (40.3%)	*n* = 0 (0%)	*n* = 45 (72.6%)
	Wales	*n* = 3 (4.8%)	*n* = 0 (0%)	*n* = 2 (3.2%)	*n* = 5 (8.1%)
	Scotland	*n* = 3 (4.8%)	*n* = 1 (1.6%)	*n* = 0 (0%)	*n* = 4 (6.5%)
	Northern Ireland	*n* = 8 (12.9%)	*n* = 0 (0%)	*n* = 0 (0%)	*n* = 8 (12.9%)
	Total	*n* = 34 (54.8%)	*n* = 26 (41.9%)	*n* = 2 (3.2%)	*n* = 62 (100%)
Gender identification	Male	*n* = 2 (3.2%)	*n* = 3 (4.8%)	*n* = 0 (0%)	*n* = 5 (8.1%)
	Female	*n* = 32 (51.6%)	*n* = 21 (33.9%)	*n* = 2 (3.2%)	*n* = 55 (88.7%)
	Non‐binary	*n* = 0 (0%)	*n* = 2 (3.2%)	*n* = 0 (0%)	*n* = 2 (3.2%)

### 3.2 Student attitudes and perceptions towards balancing environmental sustainability with person‐centred care

In relation to personal concerns about climate change, descriptive results showed that *n* = 8 respondents (12.9%) were only a little concerned or not concerned at all. The remainder of the respondents reported being somewhat (*n* = 16; 25.8%), very (*n* = 29; 46.8%) or extremely concerned (*n* = 9; 14.5%) about climate change.

Participants were asked to order the extent of their concerns about six key environmental issues derived from the Youth Climate Justice Survey (*global warming, loss of natural habitats, loss of species, climate justice, fossil fuel reduction, waste reduction*). Ranking occurred on a scale of 1–6, with 1 being the most urgent and 6 the least. The item ranked with the highest level of urgency was climate justice (M[SD] = 3.11[1.87]), followed by loss of natural habitat (M[SD] = 3.13[1.50]), global warming (M[SD] = 3.18[1.88]), loss of species (M[SD] = 3.63[1.52]), reducing waste (M[SD] = 3.71[1.66]), and fossil fuel reduction (M[SD] = 4.24[1.55]).

**FIGURE 1:**
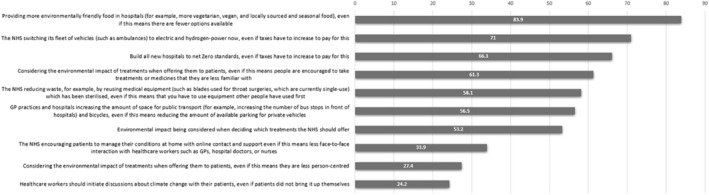
Levels of support for proposed actions.

**FIGURE 2:**
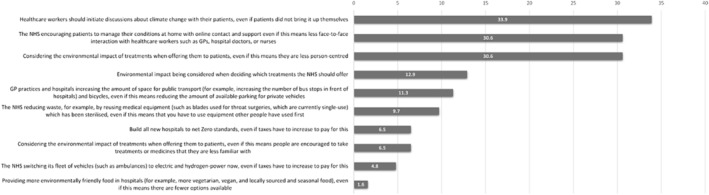
Levels of opposition for proposed actions.

Participants were also asked about their level of support for/opposition to actions being introduced in health‐care services in the *Public Perceptions of Climate Change and Health questionnaire* by The Health Foundation (2021). The responses of ‘neither support nor oppose’ and ‘do not know’ for each item were grouped together under the heading of ambivalence. Items were then ranked according to percentage levels of support for the proposed actions (Figure 1) and percentage level of opposition to the proposed actions (Figure 2).

Inherent to the structure of the action items was an element of potential personal and service‐user impacts and sacrifice through the introduction of environmental sustainability initiatives. Despite this, all of the action items were supported by at least 24% (*n* = 15) of respondents, with some reaching up to 83.9% (*n* = 52) support. Complementing this, most of the items were opposed at comparably lower levels (12.9% and below).

When interrogating the nature of the above‐mentioned action items, the top three opposed actions (i.e. three least supported) appeared to be those with potential risks to profession‐specific person‐centredness. Approximately a third of the respondents disagreed with actions that jeopardise service‐user autonomy or service access, even if such actions had the potential for environmental benefit beyond the individual. This was true in both the face of potential personal impacts and sacrifice for service users.

Participants were asked if the promotion of environmental sustainability is in some way at odds with the concept of person‐centred care; 82.3% (*n* = 51) of participants viewed sustainable practice as introducing challenges to person‐centred care. Two questions were posed related to the challenge of balancing the inherent contradiction between person‐centred care and environmental sustainability. Participants were asked if they would be willing to prioritise environmental impact considerations even if these came on account of person‐centredness. Responses varied, with 27.4% (*n* = 17) noting that they would prioritise environmental impact considerations, while 30.6% (*n* = 19) noted that they would prioritise person‐centred care. The remainder of the sample (41.9%; *n* = 26) was ambivalent. Additionally, participants were asked their views on whether issues of environmental sustainability should be introduced into therapeutic interactions by health‐care professionals, even if the service user does not initiate such discussions. This was only supported by 24.2% (*n* = 15) of respondents, while it was opposed by 33.9% (*n* = 21) (41.9% [*n* = 26] were ambivalent).

### 3.3 Student self‐perceived preparedness for sustainable occupational therapy practice and advocacy

#### 3.3.1 Contribution of educational experiences towards occupational therapy students' sense of preparedness for environmentally sustainable practice

Respondents were asked if they felt prepared by their education on the topic of environmental sustainability. Students [18% (*n* = 11)] strongly or somewhat agreed, with the remaining majority either being ambivalent (32.3%, *n* = 20) or disagreeing to varying degrees (40%, *n* = 32). This distribution of responses was similar across undergraduate (*n* = 34) (3% somewhat agree; 97% ambivalent/disagree) and masters (*n* = 26) levels separately (8% somewhat agree; 92% ambivalent/disagree). Only 16.1% (*n* = 10) participants identified ‘school and college’ as a source of information about climate change.

Participants were also asked to report on the extent to which they feel that their occupational therapy education has prepared them for meeting challenges related to sustainable person‐centred practice. Only 21% (*n* = 13) of respondents reported that they feel prepared by their educational experiences to meet this challenge in some way, 38.7% (*n* = 24) a little prepared, 40.3% (*n* = 25) not prepared at all. No significant correlation was found between self‐reported educational readiness for sustainable clinical practice and the extent to which participants agreed with a potentially inherent discrepancy between environmental sustainability and person‐centred care (*r*[60] = −0.14; *p* = 0.28).

#### 3.3.2 Personal experiences of climate change impact in relation to self‐perceived preparedness for practice and advocacy

Most of the participants [*n* = 36 (54.8%)] indicated that they feel they have not been personally impacted by climate change. Further analysis was performed to see if the two groups experienced their occupational therapy educational preparation for sustainable health care differently; no significant differences were found in the extent of agreement with the statement regarding an inherent discrepancy between sustainability and person‐centred care, between those with self‐reported personal experience of climate change (mean rank = 29.18) and those without (mean rank = 33.41) (Mann–Whitney *U* = 411; *p* = 0.33). However, a significant difference was found in self‐perceived preparedness to meet environmental challenges between those who feel that they have been personally impacted by climate change (mean rank = 38.25) and those who do not (mean rank = 25.94) (Mann–Whitney *U* = 287; *p* < 0.01), with those self‐reporting personal experience feeling more prepared.

#### 3.3.3 Students' views on what they need for professional development as environmental sustainability occupational therapy advocates

Further categorical questions provided more insight about what participants view as facilitators and barriers towards their preparation for taking professional action on climate change, topics they desire to learn about, and where they access information. Respondents identified *documentaries and stories* (*n* = 46; 74.2%), *a love of nature* (*n* = 43; 69.4%), and *respect for the environment* (*n* = 55; 88.7%) as top facilitators of action. Conversely, the most common barriers were *feeling that no one listens* (*n* = 24; 38.7%), lack of knowledge (*n* = 21, 33.9%), *lack of information on how to get involved* (*n* = 23; 37.1%), and *feeling under‐skilled* (*n* = 23; 37.1%). The most commonly identified supports wanted were *more local opportunities to get involved* (*n* = 32; 51.6%), *more information about climate change and climate action* (*n* = 31; 50%), and *training in how to get involved in climate issues* (*n* = 24; 38.71%).

In order to support their professional development as environmental sustainability advocates, respondents noted that the topics that should most urgently be included in pre‐registration teaching are *climate justice* (*n* = 40; 64.5%), *protecting nature and biodiversity* (*n* = 37; 59.7%), and *sustainable energy* (*n* = 36; 58.1%). Finally, students were asked to highlight where they gain information about climate change, with *news sites* (*n* = 50; 80.6%), *social media* (*n* = 34; 54.8%), and *environmental organisations* (*n* = 28; 45.2%) most commonly indicated.

### 3.4 Exploring strategies for pedagogy based on students' attitudes and perceptions towards climate justice

In order to delve deeper into understanding how to meet educational needs for balancing between environmental sustainability and person‐centred care, the sample was divided into two groups for further analyse of the *Youth Climate Justice Survey* (ECO‐UNESCO, 2020) [Group A (n = 29; 46.8%): those who prioritised climate justice as an urgent action; Group B (*n* = 33; 53.2%): those who did not]. The reason for using climate justice as a factor to divide into two groups was due to the centrality of occupational justice in the attainment of wellbeing as an occupational therapy professional value. No significant differences were found for a *sense of professional responsibility of sustainable health care* or *willingness to choose pro‐environmental strategies over person‐centred care*. However, a number of statements were found to differ significantly between these groups (Table 2). Together, these statements highlighted potential unique pedagogical considerations for the two groups. For those who prioritised climate justice, key strategies for their learning could include enhancing their knowledge about sustainable living, providing a variety of multimedia information and connecting them with opportunities for actions. On the other hand, offering information, helping those who did not prioritise climate justice to engage with their friends and family as well as nature based learning opportunities might be more helpful.

**TABLE 2:** Statements from the youth climate justice survey with significant between‐group differences*

	Group A *n* = 29 (climate justice prioritised)	Group B *n* = 33 (climate justice not prioritised)	Chi‐square	Cramer's V
**Barriers**				
My friends aren't interested in climate issues	1/29 (3.4%)	8/33 (24.2%)	*χ* ^2^ = 5.38; *p* = 0.02	0.30
There's not enough information on how to get involved	4/29 (13.8%)	19/33 (57.6%)	*χ* ^2^ = 12.68; *p* < 0.001	0.45
**Enablers**				
Friends and family	11/29 (37.9%)	5/33 (15.2%)	*χ* ^2^ = 4.18; *p* = 0.04	0.26
Documentaries and stories	25/29 (86.2%)	21/33 (63.6%)	*χ* ^2^ = 4.11; *p* = 0.04	0.26
Love of nature	16/29 (55.2%)	28/33 (84.8%)	*χ* ^2^ = 6.60; *p* = 0.01	0.33
**Topics desired to learn more about**				
Climate justice (protecting the most vulnerable people from climate change)	27/29 (93.1%)	13/33 (39.4%)	*χ* ^2^ = 19.5; *p* < .001	0.56
Fast fashion	15/29 (51.7%)	9/33 (27.3%)	*χ* ^2^ = 3.89; *p* = 0.05	0.25
Sustainable food options	19/29 (65.5%)	13/33 (39.4%)	*χ* ^2^ = 4.22; *p* = 0.04	0.26
Sustainable buildings	16/29 (55.2%)	9/33 (27.3%)	*χ* ^2^ = 4.99; *p* = 0.02	0.28
Circular economy	15/29 (51.7%)	7/33 (21.2%)	*χ* ^2^ = 6.28; *p* = .01	0.32
**Support needed**				
More local opportunities to get involved in climate issues	19/29 (65.5%)	13/33 (39.4%)	*χ* ^2^ = 4.22; *p* = 0.04	0.26
Opportunities to get to know more young climate activists	10/29 (34.5%)	3/33 (9.1%)	*χ* ^2^ = 6.01; *p* = .01	0.31
**Sources accessed for information about climate change**				
Podcasts	10/29 (34.5%)	3/33 (9.1%)	*χ* ^2^ = 6.01; *p* = .014	0.31
Public figures	12/29 (41.4%)	3/33 (9.1%)	*χ* ^2^ = 8.77; *p* = .003	0.38
School and college	8/29 (27.6%)	2/33 (6.1%)	*χ* ^2^ = 5.29; *p* = .021	0.29

* Between participants who did and did not prioritise climate justice.

Qualitative content analysis of participants' text responses revealed the educational content experienced and desired on the topic of sustainability through the prevalence of particular words, concepts and terms. Only *n* = 10 (16.1%) participants responded to the question about educational experiences related to environmental sustainability; of these, one response stated that no university‐based content had been experienced. From the text of the remaining *n* = 9 written responses, 231 words of text were contributed. The relatively limited amount of text response for this question meant that the responses were simply summarised. Contents that respondents listed as having studied in their courses were definitions of sustainability, sustainability in relation to occupational justice, application of sustainability to occupational therapy practice, ways of living more sustainably (transportation, environmental impacts of single‐use health equipment, man‐made drugs and fast fashion), and the basics of global warming.

**FIGURE 3:**
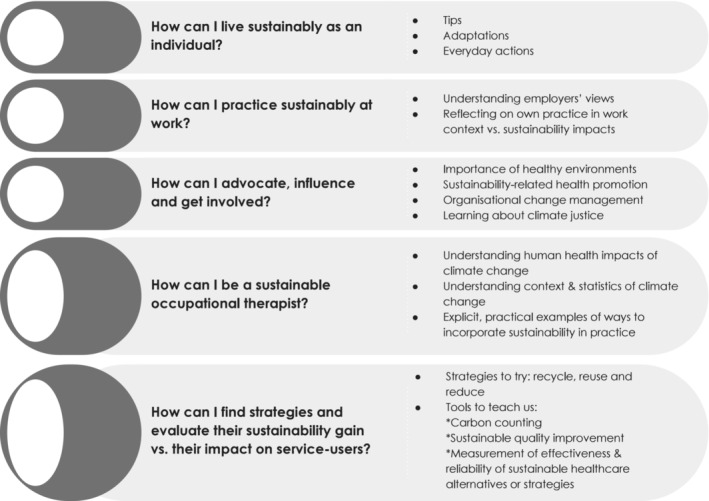
Desired learning content: How to enable sustainable occupational therapy practice.

However, with regard to the text‐based question asking students about the content that they would like to see included in their occupational therapy curricula, *n* = 43 (69.4%) provided written responses. In total, this resulted in 746 words which were for analysis. At the level of units of meaning (Elo & Kyngas, 2008), both ‘WHAT’ and ‘HOW’ units were evident in the responses. Within both these units of meaning, specific categories of coding were evident. In relation to the WHAT unit of meaning, uncertainty about what should be taught was expressed by *n* = 10 respondents, while *n* = 8 participants would welcome anything and *n* = 7 wanted more variety, diversity, depth, and degree of focus on environmental sustainability in their education. The responses provided by *n* = 22 participants related to the HOW unit of meaning; these responses were categorised under five coding categories (Figure 3).

### 3.5 Personal actions

The first of these categories focused on the more personal actions that an individual can take (*How can I live sustainably as an individual?*) and could be argued to relate to the foundational link between personal and professional identity. A quote that illustrated the desire for more personal action from participants was ‘[wanting to learn about] benefits of taking simple everyday actions. Instead of consequences of not doing enough’.

### 3.6 Daily occupational therapy practices

Three of the categories were related to content supporting actions needed in day‐to‐day occupational therapy practice, with the potential to support the development of environmentally reflective and critical occupational therapists (*How can I practice sustainably at work?, How can I be a sustainable occupational therapist?* and *How can I find strategies and evaluate their sustainability gain* vs. *their impact on service‐users?*). Quotes from participants were related to desire to learn how to practice occupational therapy sustainably day‐to‐day are, for example, ‘[teach us] strategies on how to apply it [environmental sustainability] into practice and how to gather information/generate conversation with clients/patients’ and ‘…I'd like to know if reusing/recycling them [equipment] would actually be cost effective or sustainable or if the sterilising/manufacturing processes needed to do this would actually be worse for the environment …. there needs to be [a] balance between what is effective/appropriate for each person’.

### 3.7 Wider professional responsibility

The final category related to wider professional responsibility (*How can I advocate, influence and get involved?*). Two quotes from participants illustrated their interests for influence beyond the day‐to‐day occupational therapy practices, ‘The importance of green spaces, supporting small businesses, how to advocate for change within the NHS for different equipment or ways in which services are delivered’ and ‘[how] climate injustice could be linked to occupational injustice …’.

## 4 DISCUSSION

Climate change threatens the environments in which person‐centred occupational therapy occurs, and is thus directly linked with the health, wellbeing, and occupations of current and future generations (Campbell‐Lendrum et al., 2023). From an environmental sustainability perspective, occupational therapy students are the practitioners of an uncertain future. This study aimed to explore occupational therapy students' attitudes to, perceptions of, and self‐perceived preparedness for delivering environmentally sustainable person‐centred care and advocacy and activism related to this approach to practise.

The key study findings highlighted a high level of awareness of the inequitable, disproportionate intergenerational injustice (Drolet et al., 2020) caused by climate change. Students appear to be willing to take personal actions and to advocate for changes to service provision to some degree. However, findings suggested that as specific actions led to a perceived increase of impacts on person‐centred care, support for pro‐environmental sustainability measures decreased (Figure 1). This misalignment between ideal and actual actions may be explained by incongruence between participants' espouse theory (theories individuals claim to follow) and their theory in use (theories that are inferred by actions taken) (Argyris et al., 1985). The ambiguity of how to extend one's willingness to act from the personal to the professional arena, with minimal impact on client care, represents a challenging dichotomy — and professional dilemma — between caring for persons and caring for the environment. The optimisation of how person, environment, and occupation interact is fundamental to occupational therapy (WFOT, 2016); occupational therapists of the future need to develop skills that allow them to simultaneously deliver person‐centred care, while ensuring that it is not environmentally detrimental and does not lead to occupational injustice. Findings from this study have led to the proposal of three pedagogical actions and foci that may help with closing the congruence gap between intention and action for future practitioners' professional reasoning and clinical practice, namely *specific knowledge and strategy learning, development of explicit advocacy Skills*, and *development of explicit activism skills*.

### 4.1 Environmental sustainable specific knowledge and strategy learning

Seville et al. (2023) highlighted the importance of contextualising education for sustainable practice in occupational therapy. In this study, participants suggested learning content related to personal and professional actions, including advocacy, that they felt would enhance environmental sustainability in occupational therapy practice (Figure 3). Closer examination of the responses revealed that participants want support with strategies to evaluate sustainability gain versus impact on service‐users. The desired critical evaluative skills above is an example of ‘head’ skills from the 3H model. The 3H model of holistic education claims that ‘teaching should be a unity of the head, heart and hands, that is, a unity of the cognitive, affective and psychomotor domains of learning’ (Žižanović, 2013, p.71). Participant suggestions represent predominantly ‘head’ and ‘hand’ skills within this model, and are addressed by some of the suggestions offered in Figure 3.

It is, however, the authors' contention that providing strategies for the remaining ‘heart’ element has the potential to reduce the gap between ‘espouse theory’ and ‘theory in action’ (Argyris et al., 1985) for occupational therapy environmental sustainability education and practice. Critical consideration of the centrality of the individual in occupational therapy practice models may lead to culturally‐derived concepts of self‐moderation, which may help close the gap towards greater environmental sustainability. This move away from individual‐centric views is critical as higher ecological footprints, related to higher engagement in unsustainable occupations, are evident in more individualistic countries (Hess & Rihtman, 2023; Komatsu et al., 2019).

Further tailoring of teaching strategies is worth considering in relation to findings regarding participant prioritisation of climate justice. The current study suggests that there were significant differences between those who prioritised climate justice as opposed to those who did not, with those who prioritised climate justice demonstrating a significantly higher desire to learn more about this issue as compared to those who did not. It is not surprising that those who see climate justice as important are more interested to learn about it. However, for those who did not appear to prioritise this, there nonetheless remains a responsibility for educational programmes to provide them with effective strategies to embed environmental sustainability within their future practice. This is due to the responsibility of educational programmes to develop practitioners capable of addressing occupational injustice (Drolet et al., 2020), combined with the fact that intergenerational climate‐based health impacts and occupational injustice are attributable to human activities (Sultana, 2022).

It is also worth considering the finding in this study that friends and family emerged as significant people with the power to either encourage or hinder action. For students who may not prioritise climate justice, the additional inclusion of sharing more information coupled with the use of nature as a learning environment may influence this group positively towards taking action as ‘love of nature’ was reported to be a major enabler for action (as seen in Table 2). This strategy targets a precursor for environmental concerns and behaviours called ‘nature connectedness’. Krasny (2020) defined ‘nature connectedness’ as a feeling of connection and belonging to the natural community. Contrastingly, for students who already prioritise climate justice, addressing their learning needs appears to centre around the provision of greater practical support for both individual lifestyle and collective environmental actions (ranging from the personal level of occupation of what one eats or wears, how they spend money, or where they live, to seeking local activism and advocacy opportunities) (Krasny, 2020).

### 4.2 Explicit advocacy and activism skills as a bridge for enhanced professional action

Critical reflection reveals that some content suggestions from participants (Figure 3) would require the capacity to flexibly combine head, hand, and heart skills in action (Žižanović, 2013). Notably, the categories of ‘how to advocate, influence and get involved’ and ‘how to be a sustainable allied health professional at work’ represent a continuum of development from self‐awareness through reflection, to understanding and being capable of influencing workplaces, to taking action for climate justice (referred to as ‘sustainable competency’ in WFOT, 2018). This development could also be seen as an explicit example of the development of occupational therapists as advocates and activists (Pollard & Sakellariou, 2014). Eva Lewis, a young US advocate, suggested a simple comparison of the two interrelated yet distinctively different roles: ‘To be an activist is to speak. To be an advocate is to listen’ (Soken‐Huberty, n.d.). Using principles of skill acquisition from novice to expert (Rouse & Dreyfus, 2021) and the 3H model of holistic education (Žižanović, 2013), recommendations for teaching advocacy and activist skills are described below. It is indeed important that — on the personal level — occupational therapists refrain from enforcing their individual beliefs on others. However, there remains a responsibility for occupational therapy educational programmes to impart up‐to‐date knowledge and skills about the health and wellbeing impacts of climate change and environmental sustainability, responsibilities for professional advocacy (Kirsh, 2015), and professional discussions regarding the political nature of the occupational therapy profession (Pollard & Sakellariou, 2014). Moreover, as the concept of environment is so central to the occupational therapy professional domain (American Occupational Therapy Association, 2020) (most occupational therapists would recognise claims about the foundational contribution of the person/environment/occupation paradigm), it could be argued that the occupational therapy profession has a unique responsibility for leading change in this area.

#### 4.2.1 Development of explicit advocacy skills

**FIGURE 4:**
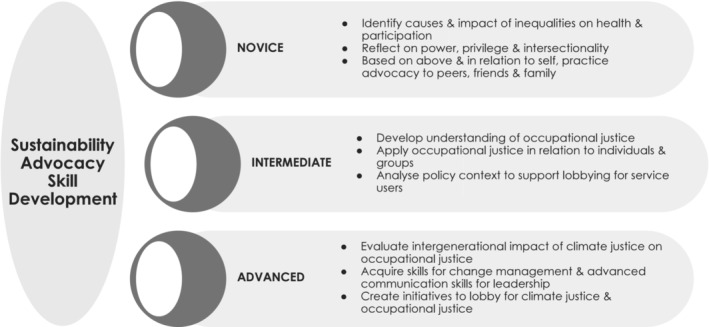
Skill development strategies for occupational therapy sustainability advocacy.

Advocacy in occupational therapy involves taking actions that allow change to occur so that clients are able to engage in occupations that meet their needs, human rights, and enhance quality of life (Dhillon et al., 2010). Occupational therapists use advocacy both for themselves (for personal fulfilment and influence) and for their clients (to ensure meeting of needs and human rights in the context of wellbeing and person‐centredness) (Dhillon et al., 2010). The learning journey of advocacy begins with valuing it as a fundamental responsibility of occupational therapy professionals. It requires learning how to listen, make arguments based on data, articulate complexity, and work within systems (Arminio et al., 2021; Dhillon et al., 2010). The developmental stages of environmental sustainability advocacy skills for occupational therapy practice start with critical self‐awareness of power, privilege, and disadvantage. This is followed by gaining skills in analysing policies as preparation for considering occupational in/justice with service users. Finally, learners need to become able to respond to intergenerational impacts of climate injustice on health and participation (Drolet et al., 2020) through visible action. Figure 4 illustrates these developmental stages, with examples of key concepts to embed at different stages across the occupational therapy curriculum.

Personal experiences were identified from the current study as a contributor to self‐perceived preparedness for environmental sustainability advocacy. Practical learning activities to develop skills for communicating with peers, friends, and family are suggested as key teaching and learning exercises, since these individuals were found to have pivotal influence for learners in relation to environmental sustainability advocacy and action. The practical activity to learn communication skills noted above should be supplemented with factual knowledge related to the health implications of climate change and unequal occupational outcomes. In addition, the creation of safe spaces to share personal experiences between students and staff should be encouraged since doing so is essential to learning about advocacy (Arminio et al., 2021). While students can share their experiences of how climate change may have impacted them or strategies they have used (self‐advocacy), staff members can share their experiences of how to manage potential conflicts and use of ‘underground practice’ as advocates (Dhillon et al., 2010). In so doing, participatory citizenship in occupational therapy education can be initiated through all parties starting to share experience and power (Kiepek et al., 2022). In turn, this should promote a sense of psychological safety and equity as students experience the potential consequences of advocacy and activism first hand.

#### 4.2.2 Development of explicit activism skills

Activism is ‘the use of direct and noticeable action beyond [that which is] conventional or routine’ (Hart et al., 2021: p1). Justice as a core occupational therapy value is translated into practice when occupational therapists act as activists (Bailliard et al., 2020; Kirsh, 2015; Pollard & Sakellariou, 2014). Kirsh (2015) argued that the occupational therapy profession must become more socially and politically responsible for the goal of enabling meaningful occupation for all. Kirsh (2015) highlighted equality and occupational justice as a global professional challenge in a general sense. However, her call for action is applicable to environmental sustainability in occupational therapy as it takes on the widest possible meaning of social and political context across space and time. The development of an activist identity requires education, combined with developing awareness that leads to moral discomfort (Hart et al., 2021). Activists can be sustained through development of resilience to resistance and commitment for action. According to Hart et al. (2021), students are more likely to challenge the status quo as activists. Therefore, a combination of experiential learning combined with guided enquiry as well as skills for the development of resilience, is suggested as a way for students to learn about activism in a supported and manageable manner (Pollard & Sakellariou, 2014).

### 4.3 Limitations

The current lack of evidence surrounding environmental sustainability in occupational therapy means that high‐quality, up‐to‐date research is vital for promoting action. Within this, student voices are powerful in helping develop effective university education, but due to the inequitable global impacts of climate change, more cross‐cultural research is needed. In the current study, the student demographic was limited to UK‐based universities which potentially presented a Western bias. Additionally, the sampling methods may have attracted students with existing interest in or concern for the topic to participate, thereby presenting a bias in results.

A further limitation lies in the fact that the surveys upon which this study was based were not developed or validated specifically for occupational therapy students; studies which employ questionnaires that are not validated for the target population may be subject to measurement error (Dowrick et al., 2015). However, the published instruments that were selected for this study provided a suitable foundation due to their relevance to health‐care practitioners, while the descriptive questions developed for the purposes of this study helped to increase the relevance and applicability of the survey to occupational therapy students. Although conclusions drawn from studies that use adapted or non‐validated questionnaires may not be made with certainty (Dowrick et al., 2015), the use of instruments selected for their broad relevance can be viewed as appropriate for exploratory research, which may be viewed as either a problem that has not been researched before, or the exploration of an existing problem to identify new ideas which cannot yet be verified (Swedberg, 2020).

The urgency of the climate crisis means that it is imperative health professionals continue engaging in research in this topic, to be transformed into knowledge for action and tangible change. This cross‐sectional exploratory study gathered data at a single time point. Longitudinal research designs may provide useful insight into student attitudes, knowledge, and action changes over the duration of their education, which has the potential to establish the efficacy of the strategies proposed here.

## 5 CONCLUSION

Addressing the urgency of the climate crisis is impeded by the challenges of the time‐lag of transferring theory and research into practical action. As the next generation of occupational therapists, students are a key demographic through which better understanding of current attitudes and perceptions with the intention of tackling this crisis can be developed. A student‐centric research approach has the potential to support knowledge translation for those people most likely to become active agents of change for the climate crisis that is imperative within the health‐care sector. Findings from this study reinforce the importance for formal occupational therapy education programmes to support students to translate their evident concern for climate change and willingness for action into knowledge, strategies, and action. For this to be achieved, occupational therapy practitioners and educators are encouraged to engage in continuing professional development opportunities to develop specific knowledge and strategies, and to reflect on where and how their skills in advocacy can be applied to environmental sustainability in occupational therapy. Equally, students must develop skills for tackling the potentially ethical dilemma, and apparent dichotomy, between meeting professional responsibilities for person‐centred care while promoting justice and equity within planetary boundaries.

## AUTHOR CONTRIBUTIONS

Felicity Murray was a pre‐registration MSc occupational therapy student at the time of the study. Ka Yan Hess and Dr Tanya Rihtman were her principal and supporting research supervisors respectively. All persons named as authors in this manuscript participated in the planning, design, and implementation of the study, and in the interpretation of the results. All persons named as authors reviewed the manuscript, approved the final version, and consented to its publication.

## CONFLICT OF INTEREST STATEMENT

The authors declare no conflicts of interest.

## Data Availability

The data that support the findings of this study are available from the corresponding author upon reasonable request.
